# Prenatal Exposure to Innately Preferred D-Limonene and Trans-Anethole Does Not Overcome Innate Aversion to Eucalyptol, *Affecting* Growth Performance of Weanling Piglets

**DOI:** 10.3390/ani11072062

**Published:** 2021-07-10

**Authors:** David Reyes-Camacho, José F. Pérez, Ester Vinyeta, Tobias Aumiller, Jan D. Van der Klis, David Solà-Oriol

**Affiliations:** 1 Animal Nutrition and Welfare Service, Department of Animal and Food Sciences, Autonomous University of Barcelona, 08193 Bellaterra, Spain; David.Reyes@uab.cat (D.R.-C.); JoseFrancisco.Perez@uab.cat (J.F.P.); 2 Delacon Biotechnik GmbH, 4209 Engerwitzdorf, Austria; evinyeta@gmail.com (E.V.); tobias.aumiller@delacon.com (T.A.); jdvdklis@gmail.com (J.D.V.d.K.)

**Keywords:** botanical compounds, weanling piglets, innate feed preference, sensory maternal learning, growth performance, maternal transfer, hyperprolific sows

## Abstract

**Simple Summary:**

Weanling piglets appear to be poorly adapted and motivated to ingest solid feed due to the innate reluctance of young animals to ingest an unfamiliar feed or flavor, i.e., feed neophobia, which commonly results in a period of underfeeding. This, and other common wean stress factors, lead to gastrointestinal disorders and impaired growth performance. Increasing the preference or familiarity for a certain type of food or for specific flavors may improve voluntary feed intake in weanling piglets. Botanical compounds (BCs) are described as functional feed additives and include sensorial properties that are able to influence feed intake and growth in pigs by dietary supplementation or sensory maternal learning. In this study, the effects of BCs such as D-limonene, *trans*-anethole, and eucalyptol on innate feed preference and growth performance of weanling piglets were evaluated by means of a double-choice feeding test and pre- and postnatal exposure to these compounds.

**Abstract:**

In the present research, two studies were performed to determine the effects of specific botanical compounds (BCs) on the innate feed preference and feed intake of piglets, as follows: Exp. 1 studied the innate feed preferences of post-weaning piglets using a double-choice feeding test. A total of 828 weaned piglets were distributed into 36 pens (23 pigs/pen) and assigned to three dietary pair choice feeding options (*n* = 12): unsupplemented prestarter diets (reference) versus reference plus D-limonene, *trans*-anethole, or eucalyptol. Piglets showed a preference for diets with D-limonene (53.8%) and *trans*-anethole (54.5%), and an aversion to eucalyptol (41.6%) (*p* < 0.05). Exp. 2 studied whether the prenatal and perinatal exposure to D-limonene, *trans*-anethole, and eucalyptol influences the feed intake and growth of newly-weaned piglets. Twenty-eight gestating and lactating sows were distributed into two dietary treatments (*n* = 14): unsupplemented Control diets or Control plus a blend of BCs (BBC; containing D-limonene, *trans*-anethole, and eucalyptol). D-limonene, *trans*-anethole, and eucalyptol were transferred into the placental fluid, and D-limonene and *trans*-anethole into the milk (*p* < 0.05). Furthermore, weanling piglets (*n* = 200; Control) and (*n* = 203; BBC) received the same treatment as their mothers in prestarter diets. The early response after weaning showed that piglets’ post-weaning BW gain was higher in the Control (*p* < 0.05) group than in those exposed to BBC. In conclusion, prenatal exposure to preferred D-limonene and *trans*-anethole, or familiarity to eucalyptol did not help to overcome the innate aversion to eucalyptol and its negative effect on weanling piglets’ BW.

## 1. Introduction

Farm pigs are fed nutritionally balanced diets with no choice, a practice that implies that voluntary feed intake is mostly based on nutritional needs rather than sensory profiles. However, there are critical phases in pig management, such as the post-weaning period [1], when palatability may have a great influence on feed intake. A major challenge for raising pigs is the transition from highly-digestible (liquid) milk to the less-digestible and more complex solid feed, and neophobia is an important aspect of post-weaning feed refusal [2]. Common consequences of this behavior are anorexia and undernutrition [3] until piglets are completely familiarized with their new feeding and environmental conditions. These effects on feed intake, coupled with other wean stress factors, i.e., physiological, environmental, and social, contribute to gastrointestinal and immune system dysfunctions leading to impaired health and growth [4]. Botanical compounds (BCs), such as those used in the present study, D-limonene [5], *trans*-anethole [6], and eucalyptol [7], have been reported to act as sensory feed additives that are able to influence feed preference and intake in pigs [8,9].

In addition, it has been described that young animals, including pigs, can learn about flavors based on the flavors in the maternal diet that are part of the amniotic fluid (prenatal) or milk (postnatal), which may reduce neophobia and modulate preference for similarly flavored food types at weaning [10]. For instance, prenatal exposure to anise [10], perinatal exposure (amniotic fluid and milk) to limonene, menthol, carvone [11], and anethol, cinnamaldehyde, and eugenol [12], all increased feed intake and growth of piglets after weaning. The literature has also described the influence of BCs on the mammalian chemosensory taste system by activating the transient receptor potential channels and taste receptor cells (referred to as taste and nutrient chemosensing) [13]. It has been suggested that in mammals, the sweet, umami, and salty tastes are innately preferred, whereas bitter and many sour tastes are innately rejected [14].

In the present study, it was hypothesized that different BCs may promote innate feed preference responses, either preferred or aversive, in early weaning piglets; while prenatal exposure to these compounds will help to reduce neophobia and enhance the early feed intake and growth of piglets after weaning. Thus, two studies were conducted under commercial conditions designed to assess the effects of specific dietary BCs on innate feed preference and sensory maternal learning as follows: Exp. 1 used a double-choice feeding test (DCHT) to investigate the effect of prestarter diets supplemented with D-limonene, *trans*-anethole, or eucalyptol versus unsupplemented control diets on feed preference at one week of post-weaning (d 8 to 14). Meanwhile, Exp. 2 aimed to determine the transfer (presence or absence) of D-limonene, *trans*-anethole, and eucalyptol from feed into the placental fluid and milk of hyperprolific sows supplemented with a blend of BCs (BBC) including the above-mentioned compounds. Exp. 2 also studied the influence of these compounds on the growth performance (BW) of newly-weaned piglets (d 1 to d 7).

## 2. Materials and Methods

All animal experimentation procedures were approved by the Ethics Committee of the Universitat Autònoma de Barcelona in compliance with the European Union guidelines 2010/63/EU for the care and use of animals in research (code CEEAH2788M2).

### 2.1. Exp. 1 (Double-Choice Feeding Test)

#### 2.1.1. Experimental Design, Animals, and Housing

A total of 828 male and female (50:50) weanling pigs ([Large White × Landrace] × Piétrain) were selected for use in Exp. 1. Piglets were weaned at d 21 of age and housed in the weaning unit on the same commercial farm (Farm 1). During the first post-weaning week (d 1 to 7) piglets were adapted to the new environmental conditions by being offered an unflavored commercial prestarter diet. At post-weaning day 7, weaned pigs with an initial BW of 6.11 ± 0.83 kg were blocked by sex and distributed according to BW into 36 pens (18 male pens and 18 female pens; 23 pigs/pen). Thereafter, pens were randomly assigned to three different dietary treatments (*n* = 12 replicate pens per treatment). Male and female pens were assigned equally to treatments. Each pen (3.20 m^2^) was equipped with two commercial pan feeders (Maxi hopper, Rotecna, Spain) and a nipple bowl drinker to provide ad libitum access to feed and water. The floor was completely slatted, and the temperature and ventilation rates were controlled using central and forced ventilation with an automatic cooling system.

#### 2.1.2. Feeding Programme and Dietary Treatments

The double-choice feeding test (DCHT) was performed during the second week after weaning (d 8 to 14). Before offering the new diets, all remaining feed was removed from the feeder used during the prestarter period. Two commercial pan feeders with hoppers were then placed in each pen to provide the reference diet and the assigned experimental diet. To avoid biases, both pan hopper feeders were hand-filled to ensure totally free access to both diets. In addition, to prevent side-effect bias in feeding behaviors, the position of the feeders inside the pen (right or left) was switched once at day 4. Experimental diets in the DCHT were the unsupplemented (basal) reference diet or the reference diet plus a botanical compound (BC) (D-limonene (7.50 g/kg), *trans*-anethole (12.17 g/kg), or 1,8-cineole (Eucalyptol) (9.73 g/kg) of feed, respectively with 2 kg/t premix dosage (Delacon Biotechnik GmbH, Engerwitzdorf, Austria) added as essential oils with wheat bran and wheat semolina as the carrier. For each dietary treatment, the BCs were pre-mixed with 5 kg of basal diet before being included in the mixer during the feed preparation process. All diets were formulated to meet the nutrient requirements for growth of newly-weaned piglets [15]. The composition of the prestarter basal diet used in the DCHT is presented in Table 1.

#### 2.1.3. Experimental Procedures, Data, and Sample Collection

Piglet BW was recorded at day 8 and day 14 of post-weaning. The feed intake for the reference and the experimental diets was measured in the second post-weaning week (day 8 to 14). In the present study, this was considered to be the innate response period of animals to the different sensory features of the feed (short-term preference) according to the methodology and reference periods described by Solà-Oriol et al. [16] and Roura et al. [17]. The feed intake values of each diet were expressed as described in Villagómez-Estrada et al. [18]. Briefly, feed intake per replicate pen was standardized by dividing feed intake by the average pig BW and by the number of pigs per replicate pen. Consequently, preference for the supplemented test diet relative to the reference diet was calculated as the percentage contribution of the test diet to total feed intake according to the following equation described by Solà-Oriol et al. [16]:

%PREFERENCE = Test diet intake/(Test diet intake) + (Reference diet intake) × 100

Therefore, preference values can range between 100 and 0%. A value of 50% indicates indifference with respect to the reference diet, whereas values significantly higher or lower than 50% indicate a significant preference or aversion, respectively.

#### 2.1.4. Statistical Analysis

In this study, different procedures with the SAS 9.4 (SAS Inst. Inc., Cary, NC, US) statistical package were used to analyze all of the data.

Standardized feed intake, percentage preference values, and piglet performance were analyzed with ANOVA using the MIXED procedure. The model included the fixed effects of treatment and the random effects of sex. The pen was considered the experimental unit (23 pigs/pen). Data were examined for outliers using the ROBUSTREG procedure. The percentage preference values for each experimental diet were transformed using the logit transformation log according to the following equation: Ln (Pref/(1 − Pref)) and, thereafter compared to the neutral value of 50% using a Student’s TTEST procedure. All the results are presented as least square (LS) means with their corresponding SEM considering a Tukey adjustment. Significant difference was determined at a probability *p* < 0.05 and tendencies were considered when *p*-values were between >0.05 and <0.10.

### 2.2. Exp. 2 (Sensory Maternal Learning)

#### 2.2.1. Experimental Design, Animals, and Housing

According to findings of innate feed preference for D-limonene, *trans*-anethole, and innate aversion for eucalyptol in Exp. 1, Exp. 2 was performed to study whether the sensory maternal learning could mitigate (familiarize) the innate aversion to eucalyptol of weanling pigs. Thus, Exp. 2 was performed as described below.

From farm 2, a total of 28 gilts and sows (up to parity 7) from a hyperprolific DanBred hybrid line (Landrace x Yorkshire) and their piglets (*n* = 409) were distributed by parity number and BW into two dietary treatment groups (*n* = 14 dams per treatment). After breeding, sows were fed unsupplemented control diets during gestation and lactation (Control) or Control diets supplemented with 1 kg/t of a blend of botanical compounds (Delacon Biotechnik GmbH, Engerwitzdorf, Austria) throughout the whole gestation and lactation period (BBC). The BBC contained 45 g/kg essential oil (EO) of feed with a 1 kg/t premix dosage (EO composition described in Table 2). After weaning, piglets from sows that had received the same diet were mixed together in a single group (*n* = 200; Control) and (*n* = 203; BBC), respectively, and moved to the nursery unit on the same farm in order to evaluate individual BW at weaning and growth performance during the prestarter phase (day 7) of post-weaning. Piglets were weaned at 23.82 ± 2 days of age with a mean BW of 5.35 ± 1.05 kg. Room temperature and ventilation rate were automatically controlled at approximately 24 °C using thermostatically controlled heaters and exhaust fans.

#### 2.2.2. Feeding Programme and Dietary Treatments

Control diets for each experimental period were formulated to meet or exceed nutrient requirements for DanBred sows [19], with adaptations based on Spanish recommendations for gestating and lactating sows, and for prestarter piglets [20] (Table 1). Experimental diets were Control plus 1 kg/t of BBC. Sows were fed an average of 2.65 ± 0.05 kg feed per day of the gestation diet of their corresponding treatments from service to 110 d of gestation, based on individual body condition. From 110 d of gestation and during lactation, sows were fed ad libitum. In addition, piglets received the same experimental treatments in the creep feed (as mash from lactation day 7 to weaning) and prestarter diets. The Control creep feed and prestarter diets were unsupplemented, whereas the experimental diets were Control supplemented with 1 kg/t of BBC. At weaning, all piglets from the same experimental treatment were allotted to a single large pen (1 pen 32.00 m^2^/treatment) with (*n* = 200; Control) and (*n* = 203; BBC) piglets in each pen, respectively, and received corresponding pelleted prestarter experimental treatments until day 7 of post-weaning. Each pen was equipped with a fully slatted floor, three ad libitum pan hoppers (Swing Feeder R3 Wet WTF, Rotecna, Spain) in the middle of the pen and nipple drinkers on the wall to provide ad libitum access to feed and water.

#### 2.2.3. Experimental Procedures, Data and Sample Collection

Placental fluid samples (60 mL per sow) were collected at farrowing as described by Blavi et al. [12], whereas milk samples (30 mL per sow) were collected at day 20 of lactation, both from the same subset of sows to determine maternal transfer of compounds (*n* = 12 per treatment). Samples were not filtered and were immediately chilled on-farm and stored at −20 °C until analysis. In addition, the individual piglet BW was recorded at weaning and day 7 post-weaning.

Maternal transfer of volatile BCs through placental fluid and milk was determined by solid-phase microextraction, gas chromatography-mass spectrometry based on the volatile BC profile characterized in the tested BBC as follows: Twelve milliliters of amniotic fluid or milk were placed into 20-mL sample vials containing 2 g NaCl (Supelco Inc., Bellefonte, PA, USA), and 40 µL of eugenol at 50 ppm was added as internal pattern. A HP6890 Series II gas chromatograph (Agilent Technologies, Salt Lake City, UT, USA) equipped with an electronic impact HP5973 detector (Agilent Technologies) was used for 30 min to extract volatiles and analyze the analytic content in the headspace. In addition, a Comb pal autosampler (CTC Analytics AG, Zwingen, Switzerland) was used to perform solid-phase microextraction. Injection was made in a splitless mode for 1 min at 265 °C. A TRB-WAX gas chromatographic column with the dimensions 60 m/mm length, 0.25-mm i.d., and 0.25-μm film thickness (Supelco Inc.) was used. Column flow (He) was 1.5 mL/min. Injector temperature was maintained at 100 °C for 10 min and raised to 265 °C, at 12 °C/min, for 17 min. The data were processed using data analysis (Agilent Technologies). The minimum detection limits were 16 ppb.

In addition, the control samples (without BBC supplementation) were used for the systematic analysis of samples, with 4.8 ppm of anethole to verify the instrumental sensitivity response to the tested BBC. Results for relative peak abundance were estimated based on the ratio (abundance/retention time) for each compound and expressed as a percentage of the proportional increase in concentrations in relation to the assigned reference values (basal value = 100%) in unsupplemented Control sows.

#### 2.2.4. Statistical Analysis

Each piglet was considered an experimental unit for BW evaluation. After transformation, placental and milk transfer values, as well as piglet BW were analyzed using the TTEST procedure of the SAS statistical package 9.4 (SAS Inst. Inc., Cary, NC, US). All data were analyzed considering the treatment as the main effect, and the results are presented as least square (LS) means with their corresponding SEM. Finally, mean significant differences were declared at *p* < 0.05, while 0.05 ≤ *p* < 0.10 were considered significant trends.

## 3. Results

### 3.1. Exp. 1 (Double-Choice Feeding Test)

#### 3.1.1. Innate Feed Preference

The piglets’ innate preference for the experimental diets is shown in Table 3. The results indicate a greater preference among piglets for diets supplemented with D-limonene (53.8% vs. 46.2%; *p* = 0.021) and *trans*-anethole (54.5% vs. 45.5%; *p* = 0.049), while piglets exposed to the diet supplemented with eucalyptol preferred the unsupplemented reference diet, indicating an aversion to the supplemented diet (41.6% vs. 58.4%; *p* = 0.002).

#### 3.1.2. Piglet’s Growth Performance

Although Exp. 1 was not designed to study the piglets’ performance due to the simultaneous exposure to the standard and supplemented diet, interestingly, piglets within the group exposed to D-limonene and *trans*-anethole showed a higher (*p* < 0.05) average daily gain (ADG) and total average daily feed intake (ADFI) when compared with piglets exposed to eucalyptol. In addition, the feed conversion ratio (FCR) was lower (*p* < 0.001) in piglets from the D-limonene and *trans*-anethole group than in the eucalyptol group (Table 4). Furthermore, a mortality rate (%) of 0.00 was recorded for D-limonene and *trans*-anethole, while 0.72 in the eucalyptol group (*n* = 2 dead piglets).

### 3.2. Exp. 2 (Sensory Maternal Learning)

#### 3.2.1. Placental and Milk Maternal Transfer

**Figure 1:**
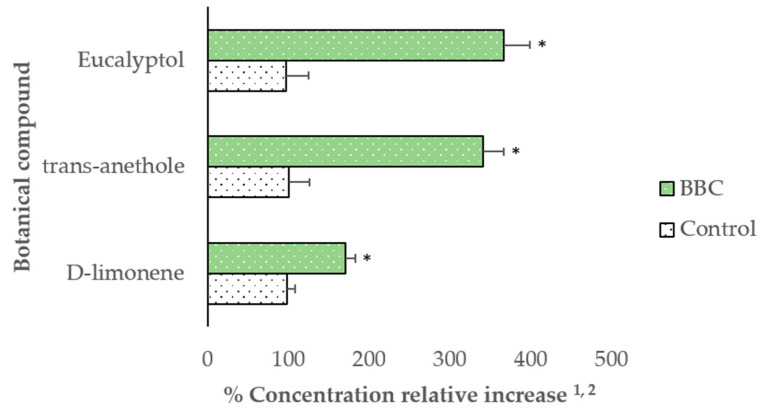
Relative concentration of botanical compounds in placental fluid of hyperprolific sows supplemented with dietary BBC. ^1^ Data are means of 12 sows per treatment (*n* = 12); ^2^ Difference in abundance/retention time of the BBC relative to assigned reference values (100%) in Control sows. Treatments: Control: unsupplemented diet; BBC: Control plus blend of botanical compounds. (*) Indicate significant differences (*p* < 0.05) using *t*-test.

**Figure 2:**
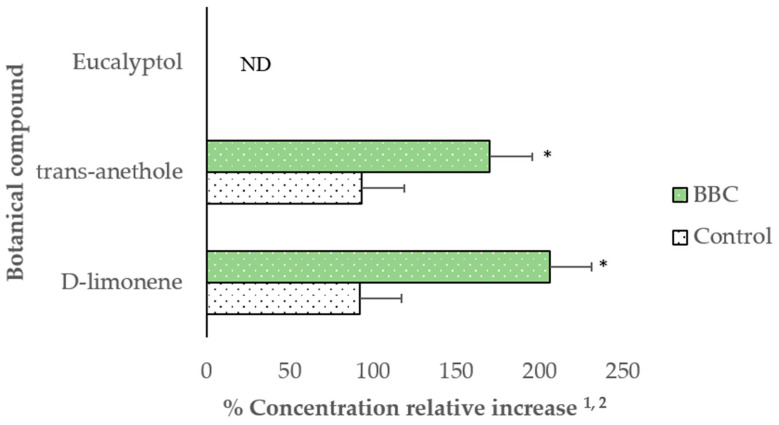
Relative concentration of botanical compounds in milk of hyperprolific sows supplemented with dietary BBC. ^1^ Data are means of 12 sows per treatment (n = 12); ^2^ Difference in abundance/retention time of the BBC relative to assigned reference values (100%) in Control sows. Treatments: Control: unsupplemented diet; BBC: Control plus blend of botanical compounds. ND: not detected. (*) Indicate significant differences (*p* < 0.05) using *t*-test.

Dietary BBC supplementation during gestation increased (*p* < 0.05) the relative concentrations of D-limonene, *trans*-anethole, and eucalyptol in sows’ placental fluid (Figure 1). During lactation, supplementation of BBC increased (*p* < 0.05) the concentrations of D-limonene and *trans*-anethole in sows’ milk, whereas eucalyptol was not detected in milk (considering a detection limit of 16 ppb) (Figure 2).

#### 3.2.2. Piglet’s Growth Performance

Regarding piglet growth performance in Exp. 2 (Table 5), there were no differences between treatments in terms of weaning piglet BW (*p* > 0.05). At post-weaning d 7, the individual piglet BW was higher (*p* = 0.037) in the Control, and the ADG decreased (*p* < 0.001) in weaning piglets from the BBC group. Moreover, a mortality rate (%) of 1.00 in the Control was recorded, while 1.93 in the BBC group (Control *n* = 2 and BBC *n* = 4 dead piglets, respectively).

## 4. Discussion

### 4.1. Innate Preference or Aversive Responses of Weaning Piglets to BCs

According to the results, weanling piglets showed significant preferences for diets supplemented with D-limonene or *trans*-anethole and an aversion to diets supplemented with eucalyptol. In addition, piglets exposed to preferred flavors of D-limonene or *trans*-anethole showed an improved growth performance (BW) when compared with exposure to the avoided eucalyptol flavor. This is rather interesting since by using the DCHT all piglets were offered an alternative option to the unsupplemented standard prestarter diet. Thus, the previous results indicate that under the conditions of this study, compared to eucalyptol, chemosensory features of the taste, such as D-limonene and *trans*-anethole, have the potential to improve the palatability and feed preferences. This would be efficient for decreasing the feed neophobia and maintaining voluntary feed intake during a feed transition, as well as growth performance (BW) in weaned piglets.

The sensorial perceptions of a feed (i.e., palatability) are mainly defined by a combination of three chemical sensations: smell (aroma), taste (flavor), and somatosensory (e.g., texture). These are important determinants for feed acceptance and later feeding behaviors [21]. Several studies have reported that certain BCs used as flavors and sensory additives have the ability to modify feed palatability and, thus, modulate feed intake and preferences in piglets [8,9,22]. However, the results are contradictory and depend on factors including additives and diet composition, inclusion rate and periods of exposure, as well as age and sex. For instance, Clouard and Val-Laillet [8] described that at postweaning d 16 (i.e., the day of feed transition), a diet supplemented with *Citrus sinensis* immediately increased palatability and acceptance of the unfamiliar starter diet, while supplementation with *Stevia rebaudiana* (stevia) and an extract of high-saponin plants increased palatability only after a few days of exposure, suggesting a long-term familiarization processes. However, no effects on feed intake and growth performance were observed. In addition, Clouard et al. [9] found that during one-way and/or two-way choice tests, one-week weaned piglets consumed the meals supplemented with extracts of *Citrus sinensis* 0.12 mL/kg, *Cinnamomum camphora L.* (camphor), *Cinnamomum aromaticum Nees* (cinnamon), and *Illicium verum* (star anise) less than the standard diet. Thus, these authors suggest that these functional ingredients in food, at these concentrations, did not improve food palatability and did not increase food intake.

Moreover, Michiels et al. [22] used a two-way choice test 10 days post-weaning, and described that, compared to the control standard diet, feed supplemented with 125, 500, 1250, and 2000 mg/kg thymol was avoided by weanling piglets. However, when feed contained 2000 mg/kg thymol plus flavor A (intense artificial sweeteners) it was preferred, but not for the feed with 2000 mg/kg thymol plus flavor B (containing flavor A plus a caramel aroma). In addition, camphor is a known TRPA1 inhibitor and hence a candidate for reducing thymol’s TRPA1 activation potential [23]. However, Michiels et al. [22] concluded that exposure to camphor (50 and 200 mg/kg) did not mitigate feed avoidance caused for thymol, thus, thymol’s bitter taste sensations might be largely responsible for feeding aversions. In this regard, the BC eucalyptol is a known TRPM8 agonist, whose activation induces a noxious cooling/burning sensation [24]. Meanwhile, TRPM5 activation by sweet tastants by an indirect mechanism causes an increase in intracellular Ca^2+^ levels due to the phytochemicals activating the sweet-taste receptor [13]. In addition, TRPM5 is specifically expressed in oral and extra-oral taste receptor cells (TRCs) such as sweet heterodimer T1R2 + T1R3, which in turn is co-expressed with glucose transporter (GLUT2) and Na^+^-dependent glucose/galactose co-transporter (SGLT1) [25], which is responsible for stimulating the secretion of glucagon-like peptide (GLP)-1 and -2 [26].

Eucalyptol can be described as having a fresh camphor-like smell and a spicy cooling taste [7] compared to the sweet citrus-like flavor and aroma of D-limonene [27] or the sweet anise-like flavor of *trans*-anethole [6]. Although it was not an aim of this study to study the effects of BCs on the chemosensory system, the results suggest that compared to eucalyptol, growth performance improvements in weanling pigs that preferred D-limonene and *trans*-anethole in the DCHT, may be related to a stimulus of voluntary feed intake due to higher appetence, nutrient absorption, and peptide secretion in the gastrointestinal tract. Studies in mammals, for instance in marsupials such as the brushtail possum (*Trichosurus vulpécula*), described that food intake is constrained in diets containing 1,8-cineole (eucalyptol) [28]. However, to our knowledge, there are no studies describing this effect on pigs.

### 4.2. Effects of Prenatal Exposure to BBC on Weaning Piglet’s Growth Performance

Oostindjer et al. [29] mentioned that pre- and postnatal exposure to flavors through the amniotic fluid and mother’s milk derived from the maternal diet has been shown to modulate food preferences and neophobia of young animals of several species. Regarding the possible biological explanations for the previous findings, it is important to highlight the role of the sensory maternal learning on the porcine nutritional programming and/or sensory conditioning during the pre- and postnatal period. Early exposure to volatile flavor components detected by the olfactory and taste systems beginning in utero and continuing during early milk feedings is considered to have a strong influence on flavor preference or aversion during weaning. Thus, pre- and postnatal experiences with food flavors transmitted from the mother’s diet provide the earliest opportunity to present specific flavors to the offspring in order to influence food acceptance and preferences [30]. In fact, sensory programming effects or familiarity impact by transfer of BCs from the sow’s diet to the amniotic fluid and/or milk have been shown particularly for BCs such as anise [29], limonene, menthol, and carvone [11], anethol, cinnamaldehyde, and eugenol [12], which influenced the feeding behaviors and growth performance of weanling piglets.

In this study, it was hypothesized that prenatal exposure of piglets to innately avoided BCs such as eucalyptol could have a favorable impact on familiarity and later acceptance. However, the present results do not support this hypothesis. Considering the results on an innate preference for D-limonene and *trans*-anethole in Exp. 1, and an innate aversion for eucalyptol, Exp. 2 aimed to study whether a sensory maternal learning by pre- and postnatal exposure to these BCs could help to mitigate the aversion and familiarize the weanling pigs to eucalyptol. According to the observations in Exp. 2, all BCs tested in the DCHT (D-limonene, *trans*-anethole, and eucalyptol) added as supplements to sow feed were transferred to the placental fluid. In contrast, only D-limonene and *trans*-anethole were detected in the milk. A possible metabolization effect for metabolites of eucalyptol that are transferred to the blood, or even metabolic activity in the milk itself [31], may be the reason for the non-transfer of eucalyptol through sows’ milk. Furthermore, these results demonstrate a potential difference in exposure of piglets before and after farrowing to selected BCs from maternal diets. Weanling piglets receiving the same BBC (containing preferred D-limonene and *trans*-anethole) in the pre-starter diet as the BBC supplemented to their mothers’ diet during gestation showed lower BW gain during the first week post-weaning compared to the Control group.

In mammals, including humans, taste begins to emerge earlier than other senses such as sight and sound. During the last trimester of prenatal development, the taste buds are already capable of detecting information and transmitting it to the central nervous system [32]. In fact, the fetus can detect pleasant and unpleasant tastes and flavors. For instance, fetal swallowing frequency increases in response to the introduction of sweet solutions to the amniotic fluid and decreases in response to the introduction of bitter solutions [30]. However, at post-weaning (first week), even though eucalyptol was transferred only prenatally from sow to offspring, no positive familiarization effect was observed. Apparently, the aversive effect of eucalyptol seems to over-ride any positive effects of preference on feed intake of D-limonene and *trans*-anethole when they were mixed in the BBC. The above observations suggest a mixture effect with the BBC where the positive response (pleasant sensations) of D-limonene and *trans*-anethole on the chemosensory system of weanling piglets could be suppressed due to the negative response (noxious sensations) to eucalyptol.

There is a well-known mixture suppression effect in gustation. Unpleasant odors and tastants are known to prevail over pleasant ones when they are mixed together [24]. For instance, Michiels et al. [22] described that in weaned piglets, camphor did not avoid aversions caused by thymol’s bitter taste. In addition, mixture-specific effects of phytochemicals, including eucalyptol and menthol, have been described for the modulation of trigeminal chemosensory systems, with an intensification of noxious sensations such as burning, cooling, and tingling, even in the absence of an olfactory percept [24]. This suggests that potential mixture effects on trigeminal stimuli are similar to the mixture effects observed for smell and taste. Therefore, the BW results for the newly-weaned piglets in Exp. 2 suggest that the pleasant flavors of D-limonene and *trans*-anethole that led to their preference in the DCHT did not mask the innate aversion to eucalyptol, which could be prenatally conditioned.

It must be concluded that one-week weanling piglets showed preferences for prestarter diets supplemented with BC such as D-limonene or *trans*-anethole, while eucalyptol supplementation led to a reduced feed acceptance. However, when the above-mentioned BCs are mixed, the apparent positive effects (pleasant odor and/or taste) of D-limonene and *trans*-anethole are not sufficient to overcome the aversion caused by eucalyptol, resulting in impaired growth performance (BW) of newly-weaned piglets. Moreover, sensory maternal learning (prenatal exposure) to eucalyptol is not sufficient to compensate for this effect. Behavior towards these types of substances may be innate and cannot be trained easily through prenatal exposure.

## Figures and Tables

**Table 1:** Ingredients and nutrient composition of diets used in Experiment 1 and 2.

Item	Experiment 1	Experiment 2		
	Piglets Prestarter	Sows’ Gestation	Sows’ Lactation	Piglets Prestarter
Ingredients, %				
White Broken Rice	60.00	-	-	-
Soybean Meal, 47% CP	28.55	2.50	13.50	5.00
Wheat	3.86	9.00	25.55	10.00
Soy Oil	3.00	-	-	-
Barley	-	35.00	10.00	15.00
Maize	-	22.70	27.01	44.00
Wheat Middling’s	-	15.00	7.00	-
Sweet Milk Whey	-	-	-	10.26
Sunflower Meal	-	5.65	4.50	-
Sugar Beet Pulp	-	3.10	2.50	-
Maize Flour	-	-	-	1.01
Soybean Protein Concentrate	-	-	-	5.00
Rapeseed Meal	-	2.50	4.50	-
Calcium Carbonate	-	0.99	1.25	-
Lard	-	1.05	1.00	-
Plasma	-	-	-	1.50
Extruded Soybean	-	-	-	5.13
Dicalcium Phosphate	2.27	0.99	1.25	0.87
Salt	0.57	0.40	0.50	0.25
L-Lysine HCl	0.56	0.31	0.63	0.81
DL-Methionine	0.29	-	-	0.23
L-Threonine	0.28	0.10	0.18	0.21
L-Valine	0.15	-	-	0.09
L-Tryptophan	0.08	-	-	0.03
Mycofix Plus 3.E	-	0.10	0.10	-
Vit-Min Premix	0.40 ^1^	0.50 ^2^	0.50 ^2^	0.60 ^3^
Calculated Nutrient Composition				
Net Energy, kcal/kg	2591	2261	2455	2438
Crude Protein, %	19.5	13.0	16.7	17.0
Calcium, %	0.70	0.85	0.91	0.30
Total Phosphorus, %	0.72	0.56	0.57	0.40
Digestible Phosphorus, %	0.38	0.35	0.37	0.30
SID Lysine, %	1.46	0.60	1.00	1.11

^1^ Supplied the following per kg of diet: 7000 IU of vitamin A (acetate); 500 IU of vitamin D3 (cholecalciferol); 250 IU of vitamin D (25-hydroxicholecalciferol); 45 mg of vitamin E; 1 mg of vitamin K3; 1.5 mg of vitamin B1; 3.5 mg of vitamin B2; 1.75 mg of vitamin B6; 0.03 mg of vitamin B12; 8.5 mg of D-pantothenic acid; 22.5 mg of niacin; 0.1 mg of biotin; 0.75 mg of folacin; 20 mg of Fe (chelate of amino acids); 2.5 mg of Cu (sulphate); 7.5 mg of Cu (chelate of glycine); 0.05 mg of Co (sulphate); 40 mg of Zn (oxide); 12.5 mg Zn (chelate of amino acids); 12.5 mg of Mn (oxide); 7.5 of Mn (chelate of glycine); 0.35 mg of I, 0.5 of Se (organic); 0.1 mg of Se (sodium); ^2^ Supplied the following per kg of diet: vitamin A (retinyl acetate), 10,000 IU; vitamin D3 (cholecalciferol), 2000 IU; vitamin E (acetate de tot-rac-3-tocopheryl), 45 mg; vitamin K3 (menadione nicotinamide bisulfite), 3 mg; vitamin B1 (thiamine mononitrate), 3 mg; vitamin B2 (riboflavin), 9 mg; vitamin B6 (pyridoxine hydrochloride), 4.5 mg; vitamin B12 (cyanocobalamin), 0.04 mg; nicotinamide, 51 mg; pantothenic acid (calcium D-pantothenate),16.5 mg; biotin (D-(+)-biotin), 0.15 mg; folic acid, 1.8 mg; choline chloride, 350 mg; iron (as iron sulphate monohydrate), 54 mg; zinc (as zinc oxide), 66 mg; manganese (as manganese oxide), 90 mg; iodine (as calcium iodine anhydrous), 1.2 mg; selenium (as sodium selenate), 0.18 mg; copper (as copper sulphate pentahydrate), 12 mg; ethoxyquin, 4 mg; D,L-malic acid, 60 mg; fumaric acid, 75 mg; sepiolite, 907 mg; vermiculite 2001 mg; colloidal silica 45 mg; ^3^ Supplied the following per kg of diet: vitamin A (retinyl acetate), 10,000 IU; vitamin D3 (cholecalciferol), 2000 IU; vitamin E (allrac α-tocopheryl-acetate) 100 ppm; choline chloride, 187 ppm; iron (as iron sulfate monohydrate), 100 ppm; iodine (potassium iodide), 100 ppm; copper (as copper sulphate pentahydrate),149 ppm; manganese (as manganese oxide), 58 ppm; zinc (as zinc oxide), 120 ppm; selenium (as sodium selenate), 0.30 ppm; selenomethionine (produced by Sac-charomyces cerevisiae), 0.1 ppm; butyl-hydroxytoluene (BHT), 63 ppm; citric acid, 8 ppm.

**Table 2:** Botanical composition of supplemented EO in the BBC ^1^.

Botanical Component	g/kg in Premix
*Trans*-Anethole	12.17
1,8-Cineole	9.73
Camphor	7.42
p-Cymene	2.56
D-Limonene	2.31
α-Terpineol	2.07
Borneol	1.83
α-Pinene	1.70
Linalool	1.46
β-Pinene	1.34

^1^ Total EO content was about 45 g/t of feed with the 1 kg/t premix dosage.

**Table 3:** Feed preference percentage of weaned piglets (d 8 to d 14) for tested BCs versus unsupplemented reference diets by using a double-choice feeding test.

Item	Treatment Groups		
	D-Limonene	*Trans*-Anethole	Eucalyptol
Feed Preference ^1,2^, %	53.8 ± 3.77	54.5 ± 5.93	41.6 ± 5.74
*p*-Value ^3^	0.021	0.049	0.002

^1^ Data are means of (*n* = 12) pens with 23 pigs per replicate pen; ^2^ Preference index significantly (*p* < 0.05) >50% indicate preference, while <50% indicate aversion using T-test; ^3^ *p*-value were calculated by using the logit transformation log.

**Table 4:** Effects of BCs on piglet’s post-weaning performance (d 8 to d 14) within test groups of a double-choice feeding test.

Item	Treatment Groups			SEM ^1^	*p*-Value
	D-Limonene	*Trans*-Anethole	Eucalyptol		
Initial BW, kg	5.86	6.14	6.32	0.300	0.558
Final BW, kg	7.44	7.86	7.31	0.369	0.529
ADG, g	225.4 ^a^	255.1 ^a^	143.0 ^b^	0.017	0.002
ADFI ^2^, g	252.9 ^a^	264.3 ^a^	213.7 ^b^	0.014	0.040
FCR	1.14 ^b^	1.05 ^b^	1.50 ^a^	0.043	<0.001

^1^ Data are means of (*n* = 12) pens per treatment with 23 pigs per replicate pen; ^2^ Values for ADFI consider combined feed intake of reference and supplemented diet; ^a,b^ Means within a row with different superscripts indicate significant differences (*p* < 0.05).

**Table 5:** Effects of supplemented BBC in prestarter diets on newly-weaned piglet’s growth performance (d 1 to 7).

Item	Treatments		SEM ^1^	*p*-Value
	Control	BBC		
Individual Piglet BW, kg				
Weaning	5.34	5.41	0.101	0.588
Post-Weaning d 7	5.43	5.14	0.098	0.037
ADG Weaning to Post-Weaning d 7	12.5	−38.9	8.932	<0.001

^1^ Data are means of (*n* = 200) and (*n* = 203) piglets for Control and BBC, respectively. Treatments: Control: unsupplemented diet; BBC: Control plus blend of botanical compounds. Statistical significance was assumed at (*p* < 0.05) using *t*-test.

## Data Availability

The data presented in this study are available on request from the corresponding author.
